# Thermal Stability and Kinetics of Formation of Magnesium Oxychloride Phase 3Mg(OH)_2_∙MgCl_2_∙8H_2_O

**DOI:** 10.3390/ma13030767

**Published:** 2020-02-07

**Authors:** Michal Lojka, Ondřej Jankovský, Adéla Jiříčková, Anna-Marie Lauermannová, Filip Antončík, David Sedmidubský, Zbyšek Pavlík, Milena Pavlíková

**Affiliations:** 1 Department of Inorganic Chemistry, Faculty of Chemical Technology, University of Chemistry and Technology, Technická 5, 166 28 Prague 6, Czech Republic; michal.lojka@vscht.cz (M.L.); ondrej.jankovsky@vscht.cz (O.J.); adela.jirickova@vscht.cz (A.J.); lauermaa@vscht.cz (A.-M.L.); filip.antoncik@vscht.cz (F.A.); david.sedmidubsky@vscht.cz (D.S.); 2 Department of Materials Engineering and Chemistry, Faculty of Civil Engineering, Czech Technical University in Prague, Thákurova 7, 166 29 Prague 6, Czech Republic; pavlikz@fsv.cvut.cz

**Keywords:** magnesium oxychloride cement, non-hydraulic binder, MOC phases, kinetics of formation, thermal stability

## Abstract

In this paper, magnesium oxychloride cement with stoichiometry 3Mg(OH)_2_∙MgCl_2_∙8H_2_O (MOC 3-1-8) was prepared and characterized. The phase composition and kinetics of formation were studied by X-ray diffraction (XRD) and Rietveld analysis of obtained diffractograms. The chemical composition was analyzed using X-ray fluorescence (XRF) and energy dispersive spectroscopy (EDS). Furthermore, scanning electron microscopy (SEM) was used to study morphology, and Fourier Transform Infrared (FT-IR) spectroscopy was also used for the analysis of the prepared sample. In addition, thermal stability was tested using simultaneous thermal analysis (STA) combined with mass spectroscopy (MS). The obtained data gave evidence of the fast formation of MOC 3-1-8, which started to precipitate rapidly. As the length of the time of ripening increased, the amount of MgO decreased, while the amount of MOC 3-1-8 increased. The fast formation of the MOC 3-1-8 phase at an ambient temperature is important for its application in the production of low-energy construction materials, which corresponds with the challenges of a sustainable building industry.

## 1. Introduction

In the past few decades, Portland cement (PC) has become a dominant material in the construction industry, putting other materials aside. These materials were historically and locally used in consideration of traditions, local conditions, and specific fields of application. It changed after the discovery of PC in the 19th century and after its expansion over the last sixty years. Nowadays, cement is the most consumed material for construction development around the world. The current cement consumption is about 4.6 billion tons annually [1,2]. Unfortunately, the production of cement is associated with an adverse environmental impact and is responsible for approximately 7% of the total anthropogenic CO_2_ emissions [3]. Moreover, cement manufacturing requires a high amount of energy for clinkering (~12% to 15% of industrial energy consumption [4]) and depletes natural resources due to the consumption of large quantities of raw materials, such as high-grade limestone and clays [5]. Therefore, taking into account the environmental sustainability issues, there is a big tendency to rediscover low-energy materials that are not widely used and have been set aside because of PC. Magnesium carbonate and reactive magnesia cements can be put into this group of materials [6,7,8,9,10]. Magnesia-based cements represent potentially CO_2_-negative materials, and, also, they can be considered eco-friendly. They have been used in practice for more than 150 years.

Magnesium oxychloride cement (MOC) is a type of non-hydraulic binder that is obtained by mixing MgO powder with the aqueous solution of magnesium dichloride. This process does not require humid curing [11]. MOC was discovered by Sorel in 1867 [12]. The reaction between MgO and MgCl_2_ provides a gel-like product that hardens after a specific curing time (depending on the content of different MOC phases) [13]. The magnesium oxide used in this reaction is obtained by the calcination of magnesite (MgCO_3_), usually at 750 °C. One of the advantages of MOC is that MgO requires a lower calcination temperature than the MgO used for PC. This causes a decrease in the use of energy [14]. Although the use of MgO-rich limestone for the production of PC is rare [15], it has been reported that MgO could lower the melting temperature, increase the quantity of the liquid phase, and change the crystal structure of the mineral phases [16,17].

The cured product, based the on the reaction of MgO and MgCl_2_ solution, can be described as a MgO–MgCl_2_–H_2_O system. Depending on the molar ratio of the three parts of this system, possible products can be “Phase 3” (3Mg(OH)_2_∙MgCl_2_∙8H_2_O) and “Phase 5” (5Mg(OH)_2_∙MgCl_2_∙8H_2_O), or “Phase 2” (2Mg(OH)_2_∙MgCl_2_∙4H_2_O) and “Phase 9” (9Mg(OH)_2_∙MgCl_2_∙5H_2_O). The presence of these phases is limited by temperature. Phase 5 and Phase 3 are present at an ambient temperature, while Phase 2 and Phase 9 are present at elevated temperatures (above 100 °C) only [18,19]. The microstructure of these reaction products shows well-crystallized materials. The crystallized needles can be described as scroll-tubular whiskers. Additional bonding of the whiskers is a major source of the mechanical strength of MOC [20]. All phases of MOC occur as well-crystallized needles that interlock in a rapid growth. If the growth is inhibited by a lack of space, crystals intergrow into a denser structure [21].

MOC has superior properties in comparison to PC. It has sufficient fire resistance [22], low thermal conductivity (comparable to PC) [23], and good resistance to abrasion [24]. An important superior property of MOC is its bonding ability, as it contributes to its high compressive strength (approximately twice as high compared to PC) [25]. Furthermore, MOC is suitable for the incorporation of organic and inorganic aggregates. This property makes it appropriate in the fixation of sewage sludge and in the solidification of municipal solid waste [26].

MOC appears stone-like and can be used in flooring [27], fire protection [28], as a material for grinding wheels [29], or as artificial ivory [30]. The curing time is much shorter than the curing time of PC, so it can be used for quick provisional repairs. [31]. Due to its external appearance, it can be used in insulation panels of rendered walls, in stuccos with revealed aggregates, and for decorative purposes—for example, in light-weight composite products with wood to create wood-like materials with better mechanical properties. The material has lower alkalinity (pH of 10–11) in comparison to ordinary cement (pH of 12–13). This property makes it suitable for usage in composites with glass fibers [32]. When used in the solidification and stabilization of sewage sludge, MgO and MgCl_2_ rapidly harden in a reaction with the water contained in the sludge and create a solid system containing two reaction phases—Phase 5 and Phase 3. During this process, a net structure is created on the condition of complete hydration [33].

Unfortunately, MOC has poor water resistance when exposed to an excessive amount of moisture, particularly at high temperatures [34]. This property can be affected by the use of various additives and a specific course of curing. These influences have already been intensively studied by Sglavo et al. [35], Xu et al. [24], and Liu et al. [36]. Chen et al. improved the water resistance through the use of tartaric acid or phosphoric acid [37]. Reaction products can also transform into magnesium carbonate–chloride due to the continuous exposure to air [38].

According to the literature, MOC 5-1-8 showed better mechanical properties in comparison to MOC 3-1-8. From a thermodynamic point of view, the coexistence of MgO and MOC 3-1-8 as composites is not possible [39]. On the other hand, composites containing MgO and MOC 5-1-8 have been intensively studied. In our contribution, we focused on phase MOC 3-1-8 in order to understand the kinetics of formation and the thermal performance of this phase, which is essential to understanding the whole MgO–MgCl_2_–H_2_O system. The kinetics of the formation of the MOC 3-1-8 phase is the crucial parameter for MOC use in the production of alternative, low-energy building materials and research of MOC-modified binders. The quantification of the phases was thoroughly investigated using Rietveld analysis. The thermal stability of the MOC 3-1-8 phase was also investigated in order to obtain information on structural changes that the tested material underwent at high temperatures.

## 2. Materials and Methods

The following chemicals were used for the mixing of MOC: MgCl_2_∙6H_2_O (>99%, Penta, Chrudim, Czech Republic); and MgO (>98%, Penta, Chrudim, Czech Republic). Deionized water (16.8 MΩ) was used for all syntheses. For the synthesis of the stoichiometric phase, the ratio 3 MgO to MgCl_2_∙6H_2_O to 5 H_2_O was used for the synthesis of MOC with the composition 3Mg(OH)_2_∙MgCl_2_∙8H_2_O (known as Phase 3, MOC 3-1-8, or also as Mg_2_(OH)_3_Cl∙4H_2_O). The samples were termed MOC 3-1-8, accordingly. For the experiment, we dissolved 19.54 g of MgCl_2_∙6H_2_O in 8.66 g of deionized water. In the next step, 11.62 g of magnesium oxide was added, and the suspension was intensively stirred for five minutes. One part of the suspension was then applied on the X-ray diffraction (XRD) holder (specimen holder rings PMMA, for D2 and D8, 8.5 mm high, sample reception Ø25 mm), and the rest of the suspension was placed into a plastic beaker for other analyses.

The formation of magnesium oxychloride cement MOC 3-1-8 is summarized in the following equation:

(1) $$3\;\mathrm{MgO}+{\mathrm{MgCl}}_{2}\cdot 6H_{2}O+5{\;H}_{2}O\to 3\mathrm{Mg}{\left(\mathrm{OH}\right)}_{2}\cdot {\mathrm{MgCl}}_{2}\cdot 8H_{2}O.$$

First, the raw materials were analyzed. The chemical and phase composition of commercially delivered MgO and MgCl_2_∙6H_2_O were tested using X-ray fluorescence (XRF) and X-ray diffraction (XRD) analyses. The details of the applied test methods and used apparatuses are introduced together with a description of the MOC tests below. According to XRF, the MgO content was 99.7 wt.%, and the starting material contained only traces of calcium. In the case of MgCl_2_∙6H_2_O, no significant impurities were detected. The purity was over 99.9 wt.%. Starting materials were also analyzed using X-ray diffraction, where only MgO (JCPDS 04-009-5447) and MgCl_2_∙6H_2_O (JCPDS 01 076-0789) were detected. XRD also confirmed the high purity of both starting materials: no impurities were detected. Diffractograms of MgO and MgCl_2_∙6H2O are shown in Figure S1 of Supplementary Materials.

**Figure 1:**
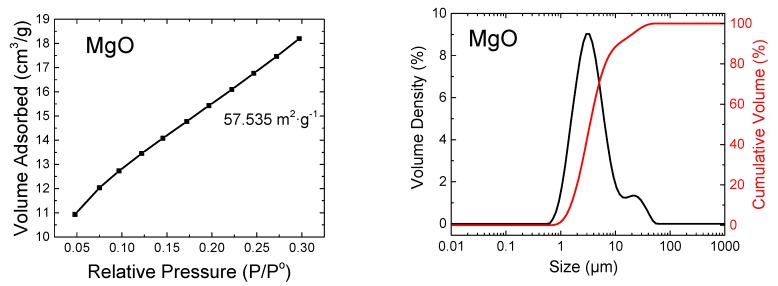
Brunauer–Emmett–Teller (BET) surface area of MgO powder (**left**) and particle size distribution of MgO cumulative and distribution curves (**right**).

In addition, for MgO powder, the Brunauer–Emmett–Teller (BET) surface area and particle size distribution were measured. The BET specific surface area was measured using a NOVAtouch LX^2^ sorption analyzer (Quantachrome Instruments, Boynton Beach, FL, USA). The sample was outgassed for 10 h at 100 °C under high vacuum. A nitrogen-cooled (77 K) detector was used for the evaluation of the results, using BET and Kelvin equations. The dry and degassed sample’s mass was 243 mg, and it used an 11-point BET measurement to obtain a more accurate isotherm. The Quantachrome software (TouchWin, Boynton Beach, FL, USA) was used for the evaluation of the measured data and to recalculate the measured value to m^2^ per 1 g of the tested sample. The obtained data are graphed in Figure 1.

The particle size distribution of commercially delivered MgO was analyzed by the laser diffraction method using a Malvern Panalytical Mastersizer 3000 device (Malvern Panalytical, Malvern, UK) with a 4 mW He-Ne 632.8 nm Red light source and a 10 mW light-emitting diode (LED) 470 nm Blue light source. The range of measurement was set from 0.01 to 1000 μm. The measurement was carried out in a wet cell using Isopropyl Alcohol (Penta, Chrudim, Czech Republic, purity p.a.). During the measurement, a constant mixing of suspension (3000 rpm) was set. The MgO was measured five times (five scans) and the particle size distribution was determined from the average values (see Figure 1).

The MOC 3-1-8 phase was analyzed using XRD, XRF, energy dispersive spectroscopy (EDS), scanning electron microscopy (SEM), and simultaneous thermal analysis–mass spectroscopy (STA-MS) methods. XRD was performed by a Bruker D2 Phaser powder diffractometer (Bruker AXS GmbH, Karlsruhe, Germany) with Bragg–Brentano geometry, applying CuKα radiation (λ = 0.15418 nm, U = 30 kV, I = 10 mA) and a rotation (five rounds per minute). The step size was set to 0.02° (2θ) and the overall data were obtained from the angular range of 5°–80°. To determine the kinetics of the formation of Phase 3, the sample was analyzed every 12 h for 7 days. X’Pert HighScore Plus software version 4.0 (PANalytical, Almelo, Netherlands) was applied to evaluate the obtained set of data.

The chemical composition of the sample was determined by XRF using an Axios sequential WD (Wavelength Dispersive)-XRF spectrometer (PANalytical, Almelo, The Netherlands) equipped with an Rh anode end-window X-ray tube fitted with a 50 μm beryllium window. The software SuperQ 5 (PANalytical) was used to collect the measured data. The sample was pressed (with no binding agents) on a pellet of H_3_BO_3_, where the total thickness was ~5 mm and the diameter was 40 mm.

SEM was used in order to study the morphology (Tescan MAIA 3, TESCAN Brno, s.r.o., Brno, Czech Republic). Using an EDS analyzer (X-Max150, Oxford instruments, High Wycombe, UK) with a 20 mm^2^ silicon drift detector (Oxford instruments, High Wycombe, UK) and AZtecEnergy software (Oxford Instruments), we determined the elemental composition and mapping. Five scans were performed with an overall measuring time of 10 min to obtain the average elemental composition. The samples were set on a conductive tape (made of carbon) to establish the conductivity of the measurements. Gold sputtering (5 nm) was used before the measurement. The electron beam for both SEM and SEM–EDS analysis was set to 10 kV, with the working distance 10 mm.

To identify the chemical environments of the inorganic bonds within the hardened MOC samples, FT-IR (Fourier Transform Infrared) spectroscopy was used. Mid-infrared spectra were analyzed on a Nicolet 6700 spectrometer (Thermo Fisher Scientific), using the ATR (attenuated total reflectance) technique after 32 scans, with wavenumbers ranging from 4000 cm^−1^ to 400 cm^−1^ and with a spectral resolution of 4 cm^−1^. Samples were prepared by homogenization in an agate grinding mortar of the samples after 28 days.

A Setsys Evolution apparatus from Setaram Instrumentation (Caluire, France) was used to carry out STA with a temperature of up to 1000 °C. The measurements were performed in a dynamic helium atmosphere, where the flow rate was set to 50 mL/min and the heating rate to 10 °C/min. The gases evolving during heating were analyzed by an OmniStar^TM^ mass spectrometer from Pffeifer Vacuum (Pffeifer Vacuum GmbH, Aßlar, Germany).

## 3. Results and Discussion

**Figure 2:**
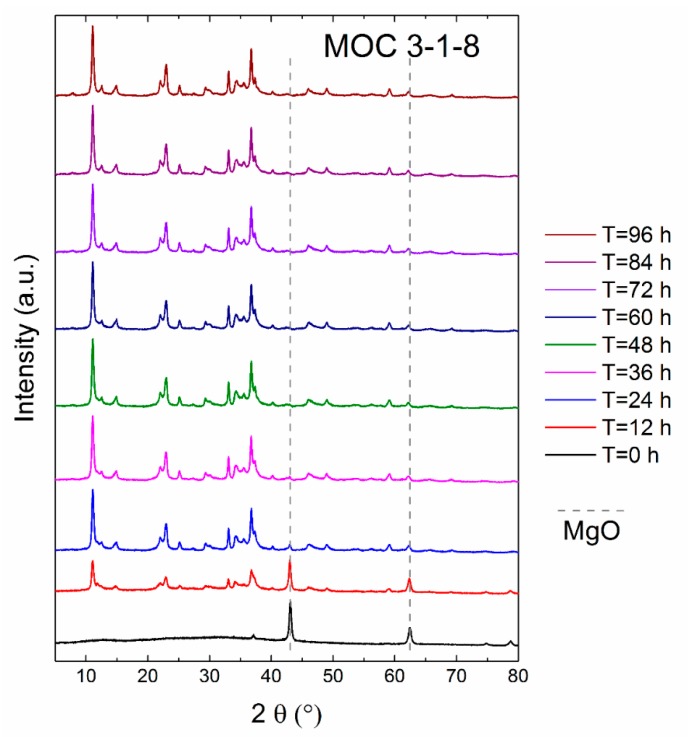
X-ray diffraction patterns of magnesium oxychloride cement (MOC) 3-1-8 suspensions measured for 96 h.

**Figure 3:**
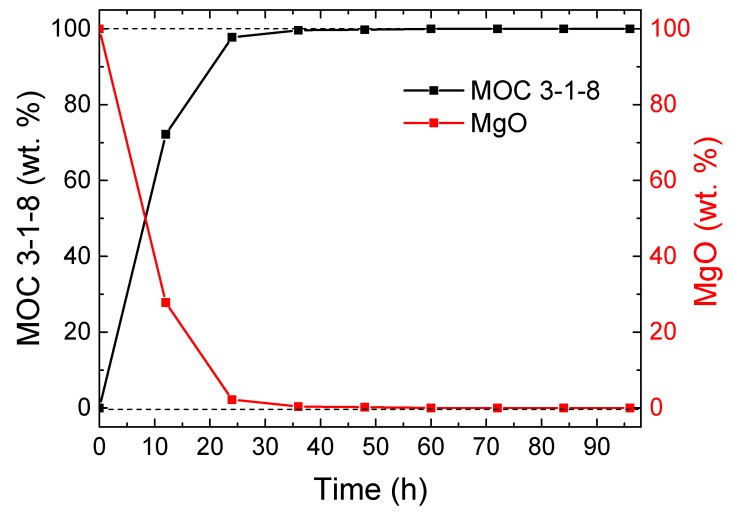
Sample composition obtained by Rietveld analysis.

In the first experiment, we tested the kinetics of formation of the phase MOC 3-1-8. XRD diffractograms were acquired every 12 h, as can be seen in Figure 2. At the beginning, only MgO (JCPDS 01-075-0447) was present in the sample because it is insoluble in water, while magnesium chloride was entirely dissolved. MgO can be seen at 2θ = 42.9 (reflection 400) and at 2θ = 62.2 (reflection 440). Note that a gray dashed line in Figure 2 shows the positions of these major reflections. Another crystalline phase, MOC 3-1-8, started to precipitate rapidly. After 12 h, the sample already contained 72% of MOC 3-1-8 (JCPDS 00-007-0412). The strongest reflection (100) for MOC-3-1-8 was found at 2θ = 11.1. With the time of ripening increasing, the amount of MgO decreased, while the amount of MOC 3-1-8 increased. Time dependence of sample’s composition is shown in Figure 3. After 36 h, the sample contained > 99 wt.% of MOC 3-1-8. Let us note that the quantification was performed for the purpose of studying the rate reaction of MgO and the formation of the MOC 3-1-8 phase; hence, only these were considered for the Rietveld refinement.

**Figure 4:**
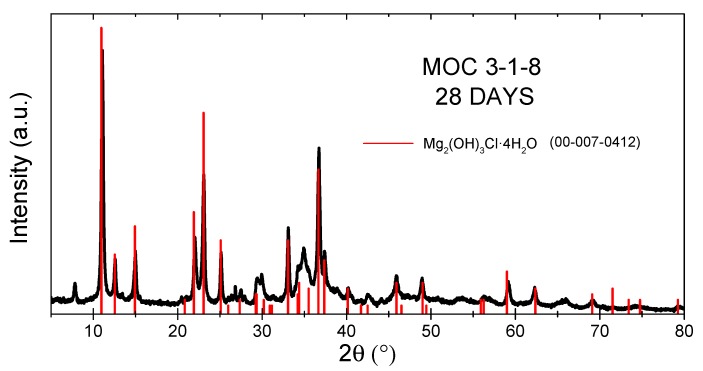
X-ray diffractogram of MOC 3-1-8 after 28 days.

Due to the environmental and financial reasons associated with the sustainability of the building industry, MOC and other low-energy, alternative materials should partially replace the construction market products based on various kinds of cement. Therefore, as ordinary Portland cement concrete is usually tested after 28 days of curing, we also measured the same MOC sample after 28 days at room temperature. The MOC 3-1-8 was detected; however, a low amount of Magnesium Carbonate Aqua Chloride Hydroxide Hydrate (Chlorartinite: JCPDS 00-061-0391) was observed on the surface (2θ = 7.8. For reflection 110, see Figure 4).

**Figure 5:**
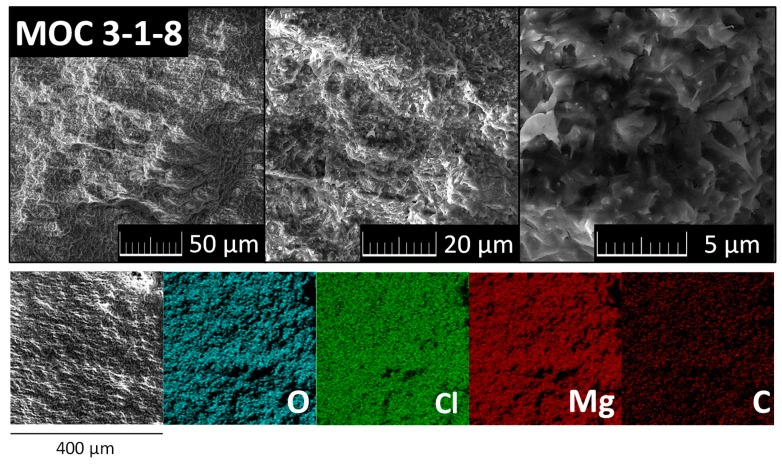
SEM micrographs and EDS elemental maps of MOC 3-1-8.

After 28 days, the chemical composition and morphology of the MOC samples were characterized using SEM and EDS. The data are shown in Figure 5. Grains with typical dimensions below 5 μm held very well together (interlocking and crosslinked crystalline products of MOC precipitation), which is typical for magnesium oxychloride cements [40]. There were no visible defects within the structure. EDS confirmed high purity, except for magnesium (19.3 wt.%), oxygen (36.9 wt.%), and chlorine (30.0 wt.%). Carbon was detected due to the presence of magnesium carbonate on the surface of the sample, and also from conductive carbon tape. The chemical composition of MOC 3-1-8 was also measured by XRF. The results show that sample MOC 3-1-8 contained 19.8 wt.% of magnesium, 29.1 wt.% of chlorine, and 51.0 wt.% of light elements such as C, O, and H (in the form of water). Sulfur and calcium were detected in very small amounts (lower than 0.05 wt.%).

**Figure 6:**
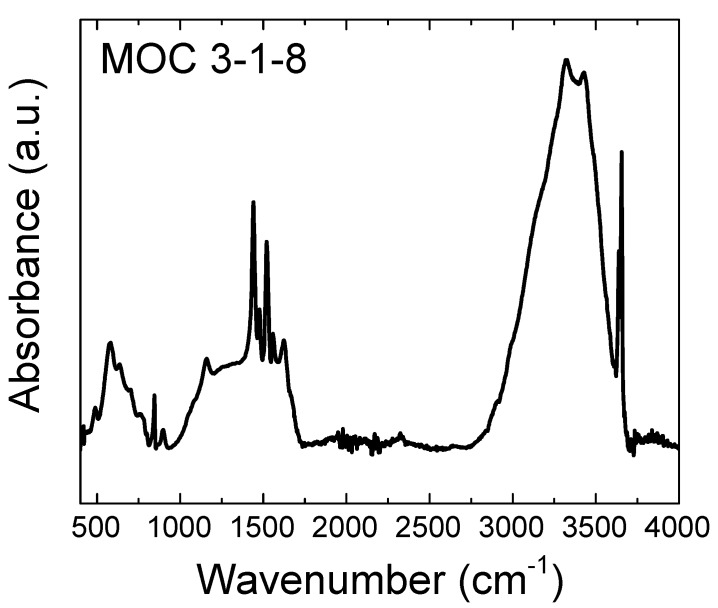
The Mid-IR spectrum of MOC in the range of 400–4000 cm^−1^.

The mid-infrared (MIR) spectrum of the MOC 3-1-8 sample is given in Figure 6. It consists of the peaks from the fundamental vibrations of structural H_2_O and the lattice vibrations of MgCl_2_ and Mg(OH)_2_ (Table 1). The peaks near 1600 cm^−1^ are due to the H_2_O bending mode vibration; the peaks in the range of 2000–3700 cm^−1^ are contributed by the symmetric and asymmetric stretching mode of O–H bonds in H_2_O and Mg(OH)_2_ [41]. The series absorption bands in the range of 1000–400 cm^−1^ correspond to the existence of lattice translation modes (Mg–OH) and vibrational modes of the lattice showing the Mg–O/Mg^2+^ and O/O–Mg–O/O–Mg^2+^–O bonds [42]. The absorption band at 845 cm^−1^ was assigned to the characteristic absorption peak of cubic Mg–O. The lattice vibration modes of Mg–O/Mg–Cl bonds appear in the absorption spectrum below 500 cm^−1^, contributed by the vibrations of the bonds of Mg–Cl or of Mg–O.

**Figure 7:**
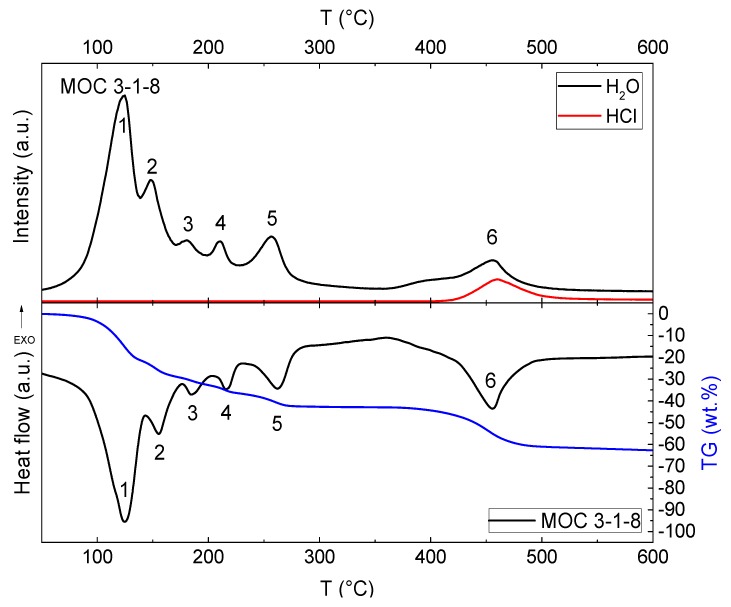
Simultaneous thermal analysis–mass spectroscopy (STA-MS) of MOC 3-1-8 measured in an inner atmosphere: MS signals for water and hydrochloric acid (**up**) and DTA plus TG signals (**down**).

Thermal stability of MOC 3-1-8 was analyzed using STA-MS (Figure 7). MS signals for water and hydrochloric acid are introduced at the top and differential thermal analysis (DTA) plus thermogravimetry (TG) signals at the bottom of Figure 7. From the data, it is obvious that after the thermal treatment, MgO was formed, and water and hydrochloric acid were released during the heating. On the DTA curve, there are six visible endothermal effects accompanied by a weight decrease. The first effect (maximum at 125 °C) was attributed to the release of four water molecules, which can be seen also from MS and TG curves (Equation (2)).

(2) $$\mathrm{STEP}\;1:\;3\mathrm{Mg}{\left(\mathrm{OH}\right)}_{2}\cdot {\mathrm{MgCl}}_{2}\cdot 8H_{2}O\to 3Mg{\left(OH\right)}_{2}\cdot MgCl_{2}\cdot 4H_{2}O+4H_{2}O$$

The second effect (maximum at 155 °C) was associated with the release of two water molecules.

(3) $$STEP\;2:\;3\mathrm{Mg}{\left(\mathrm{OH}\right)}_{2}\cdot {\mathrm{MgCl}}_{2}\cdot 4H_{2}O\to 3\mathrm{Mg}{\left(\mathrm{OH}\right)}_{2}\cdot {\mathrm{MgCl}}_{2}\cdot 2H_{2}O+2H_{2}O.$$

The third endotherm (maximum at 185 °C) was attributed to the release of one water molecule.

(4) $$STEP\;3:\;3Mg{\left(OH\right)}_{2}\cdot MgCl_{2}\cdot 2H_{2}O\to 3Mg{\left(OH\right)}_{2}\cdot MgCl_{2}\cdot H_{2}O+H_{2}O.$$

He et al. [40] published TG and differential scanning calorimetry (DSC) curves of Phase 5 of MOC. The authors observed that Phase 5 lost five molecules of water of crystallization before 200 °C; i.e., the loss in crystalline water was about two molecules of water lower than that measured for the Phase 3 analyzed in this study. The fourth endothermic effect (maximum at 215 °C) was assigned to the release of one water molecule.

(5) $$STEP\;4:\;3Mg{\left(OH\right)}_{2}\cdot MgCl_{2}\cdot H_{2}O\to 3Mg{\left(OH\right)}_{2}\cdot MgCl_{2}+H_{2}O.$$

In the next step (maximum at 265 °C), the loss of one molecule of crystalline water was also identified.

(6) $$STEP\;5:\;3Mg{\left(OH\right)}_{2}\cdot MgCl_{2}\to 2Mg\left(OH\right)Cl+Mg{\left(OH\right)}_{2}+MgO+H_{2}O.$$

The last endothermic effect (maximum at 455 °C) comprised two consequent effects, where one water molecule and two hydrochloric acids were released.

(7) $$STEP\;6:\;Mg{\left(OH\right)}_{2}\to MgO+H_{2}O$$

(8) 2Mg(OH)Cl→2MgO+2HCl.

The release of hydrochloric acid from MOC at temperatures around 400 °C was well documented by Xia et al. [33], and, later, by Dinnebier et al. [43]. However, in the presented test, the mechanism of the last step of the decomposition was described differently than reported; e.g., Cole et al. [44] and He et al. [40], who detected that only hydrochloric acid is released. We distinctly proved, using mass spectroscopy, that water was also released in this step [45].

## 4. Conclusions

The kinetics of the formation of Phase 3 of MOC and its thermal stability were studied. Although the casted samples were cured freely in a laboratory under an ambient temperature of approximately (21 ± 2) °C, the precipitation of the crystalline phase (MOC 3-1-8) started rapidly, and was almost completed after 36 h. It was verified by the measurement of MOC 3-1-8 content that it was at 36 h > 99 wt.%. The fast formation of the MOC 3-1-8 phase is very promising for the application of this binder in the production of low-energy and low-carbon materials for the construction industry—e.g., in the form of repair cements, floorings, thermal insulation boards, fiber-reinforced MOC plates, and prefabricated elements as sandwich panels, facing slabs, etc. However, one must consider the cost of pure MOC, which is about 1.5 times higher compared to that of PC. On the other hand, in the case of the use of MgO and MgCl_2_ of lower purity, the price of MOC will be significantly reduced. The prices are also affected by the volume of the production of MOC and that of Portland cement, which is much greater and thus readily available and less expensive on the construction market. Furthermore, the availability of raw materials for the calcination of MgO and PC affects the price of final commercial products. Nevertheless, the main benefit of the use of MOC cement instead of Portland cement is its low calcination temperature (approximately 700 °C) compared to the sintering temperature of PC, which is about 1450 °C. This represents great energy savings, and, thus, a lower carbon footpath. Moreover, as pure MOC phases are too expensive for practical use in the development of construction products, the possible use of industrial by-products as partial substitutes of MOC cements will be the subject of further research. The use of alternative MOC substitutes might greatly reduce the price of MOC cement-based products.

The SEM analysis revealed the dense microstructure of the MOC 3-1-8 phase, composed of interlocked and crosslinked hardened crystalline products. Based on this, the high mechanical resistance of MOC can be anticipated.

The thermal stability of the examined MOC 3-1-8 is relatively low, as it started to decompose at 125 °C and was completed at approximately 500 °C, with the total mass loss of ~63%. As the decomposition mechanisms of MOC were identified and clearly characterized, we will focus in future studies on the assessment of the effect of exposure of MOC 3-1-8 to high temperatures on its physical properties, where structural, mechanical, hygric, and thermal parameters will be of particular importance.

## Figures and Tables

**Table 1:** Assignments of the major absorption bands.

Wavenumbers (cm^−1^)	Assignment
3434	stretching modes (ν) of H–O–H in H_2_O
3654, 3638	stretching modes (ν) of O–H in Mg(OH)_2_
3319	stretching modes (ν) of H–O–H in H_2_O
2053	bending (δ) and rocking (ρ) vibrations of H–O–H in H_2_O
1608	bending (δ) vibration of H–O–H in MgCl_2_.8H_2_O
1623, 1558, 1520, 1476, 1440	stretching modes (ν) of Mg–O in MgCl_2_.8H_2_O
845	stretching vibration (ν) of the Mg–O cubic structure
634	stretching (ν) vibrations of Mg–O
581	deformation (δ) and stretching (ν) vibrations of Mg–Cl
489	translation vibrations of Mg/Mg–O, Mg–OH
417	vibrational modes of the lattice showing the Mg–O/Mg^2+^, O/O–Mg–O/O–Mg^2+^–O bonds

## Supplementary Materials

The following are available online at https://www.mdpi.com/1996-1944/13/3/767/s1, Figure S1: Diffractograms of raw materials MgO and MgCl_2_∙6H_2_O.
