# Supplementary material for: Thermal Stability and Kinetics of Formation of Magnesium Oxychloride Phase 3Mg(OH)2∙MgCl2∙8H2O
Source: Materials (Basel). 2020 Feb 7;13(3):767. doi: 10.3390/ma13030767 (PMC7040710; doi:10.3390/ma13030767)
Supplement: Supplementary file 1 [file materials-13-00767-s001.pdf]

materials

Supporting information

Thermal Stability and Kinetics of Formation of Magnesium Oxychloride Phase $3Mg(OH)_2{\cdot}MgCl_2{\cdot}8H_2O$

Michal Lojka [1], Ondřej Jankovský [1], Adéla Jiříčková [1], Anna-Marie Lauermannová [1], Filip Antončík [1], David Sedmidubský [1], Zbyšek Pavlík [2] and Milena Pavlíková [2]*

1 Department of Inorganic Chemistry, Faculty of Chemical Technology, University of Chemistry and Technology, Technická 5, 166 28 Prague 6, Czech Republic; michal.lojka@vscht.cz (M.L.), ondrej.jankovsky@vscht.cz (O.J.), adela.jirickova@vscht.cz (A.J.), lauermaa@vscht.cz (A.-M.L.), filip.antoncik@vscht.cz (F.A.), david.sedmidubsky@vscht.cz (D.S.)

2 Department of Materials Engineering and Chemistry, Faculty of Civil Engineering, Czech Technical University in Prague, Thákurova 7, 166 29 Prague 6, Czech Republic; pavlikz@fsv.cvut.cz

* Correspondence: milena.pavlikova@fsv.cvut.cz; Tel.: +420224354688

Received: 16 January 2020; Accepted: 6 February 2020; Published: date

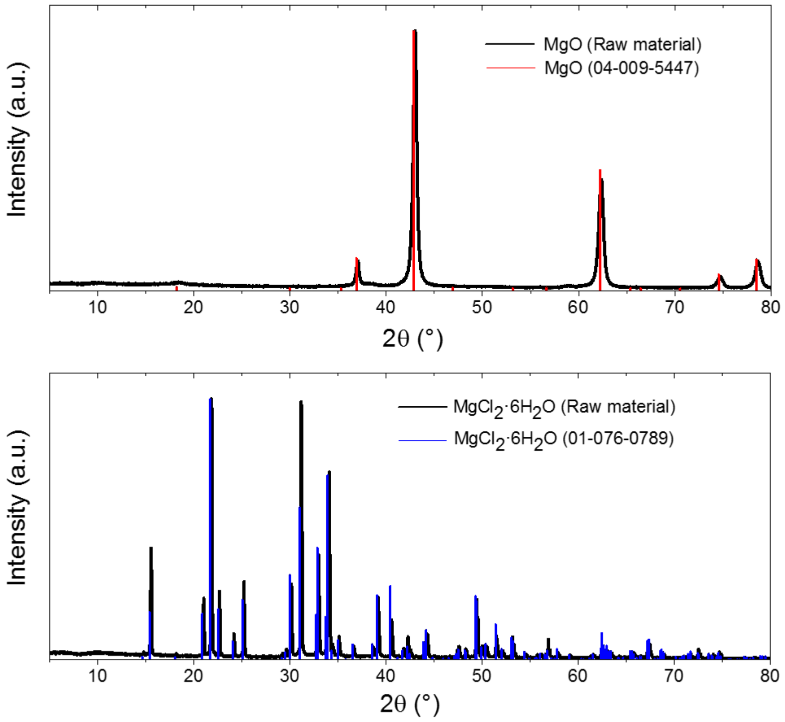

Figure S1: Diffractograms of raw materials MgO and $MgCl_2{\cdot}6H_2O$.
